# Modelling the Evolution of Social Structure

**DOI:** 10.1371/journal.pone.0158605

**Published:** 2016-07-18

**Authors:** A. G. Sutcliffe, R. I. M. Dunbar, D. Wang

**Affiliations:** 1 Manchester Business School, University of Manchester, Manchester, United Kingdom; 2 Department of Experimental Psychology, University of Oxford, Oxford, United Kingdom; 3 EBTIC, Khalifa University, Abu Dhabi, UAE; University of Lethbridge, CANADA

## Abstract

Although simple social structures are more common in animal societies, some taxa (mainly mammals) have complex, multi-level social systems, in which the levels reflect differential association. We develop a simulation model to explore the conditions under which multi-level social systems of this kind evolve. Our model focuses on the evolutionary trade-offs between foraging and social interaction, and explores the impact of alternative strategies for distributing social interaction, with fitness criteria for wellbeing, alliance formation, risk, stress and access to food resources that reward social strategies differentially. The results suggest that multi-level social structures characterised by a few strong relationships, more medium ties and large numbers of weak ties emerge only in a small part of the overall fitness landscape, namely where there are significant fitness benefits from wellbeing and alliance formation and there are high levels of social interaction. In contrast, ‘favour-the-few’ strategies are more competitive under a wide range of fitness conditions, including those producing homogeneous, single-level societies of the kind found in many birds and mammals. The simulations suggest that the development of complex, multi-level social structures of the kind found in many primates (including humans) depends on a capacity for high investment in social time, preferential social interaction strategies, high mortality risk and/or differential reproduction. These conditions are characteristic of only a few mammalian taxa.

## Introduction

It has become increasingly clear that some (but by no means all) social species live in complex, hierarchically-organised, multi-layer social systems [1–4]. In many cases, these multi-level societies are founded on bonded relationships [5–8] and, at least in primates (including humans), this ‘bondedness’ is responsible for the layering by differentiating close from weak relationships (strong vs. weak ties in the sense of [9]). Bonded relationships of this kind commonly depend on the investment of considerable time in servicing relationships, and the long term stability of such relationships is invariably fragile in the absence of such investment [3,10–16]. Perhaps not surprisingly, these kinds of multi-level social systems are relatively rare: most mammals and birds have simple, unstructured societies based on either relatively casual relationships or small social groups (e.g. monogamous pairs or harems) and only a very small number of taxa (mainly primates, elephantids, delphinids, equids) habitually exhibit multi-level sociality [2,3,7,17].

While the evolutionary origins of simple societies are well understood [18], there is no general theory (aside from kin selection) to explain the evolution of multi-level social systems, and even then none provides a principled explanation as to why these societies should be multi-layered. Why should a species invest a scarce commodity (time and/or emotional effort) into creating and maintaining a social system of such seemingly unnecessary complexity? Why do these taxa not simply form loosely organised but flexible herds like many deer and bovids, especially given the costs that bonded relationships seem to incur in terms of time investment?

One plausible explanation is that the various layers provide different benefits [19], imposing trade-offs that create sufficient viscosity to prevent individuals or sub-groups drifting completely apart. The benefits derived from close social support [20–24], for example, might promote the formation of small foraging groups or grooming cliques, while group-level benefits that derive from cooperative hunting or information exchange [18], reducing predation risk [25–28] or minimising the risks of raiding by conspecifics [4,29,30] might motivate the formation of higher-level communities.

In this paper, we use computational modelling to ask what conditions lead to the emergence of multi-level social structures in group-living species. We use humans as our test case because they provide the most explicit and best understood (as well as by far the most complex) example of a multi-level social system. Human societies are characterised by four hierarchically inclusive grouping layers that have quite specific sizes (see [31–34]), and this provides us with a quantitative benchmark for the model to match. In fact, these same layers occur in other mammal taxa, such as primates and delphinids, that have complex multi-level societies, with essentially the same numerical sizes [3]. Thus, in studying the more complex human case, not only do we maximise the complexity we have to explain in a strong test of the model, but at the same time we cover the less complex cases found in other mammalian taxa.

Human social networks and communities have been shown to consist of four separate layers of relationship [31,32,35]. The innermost two layers have been identified as the support clique with circa 5 members [36,37] and the sympathy group with 15 members [37,38], followed by an affinity group of 50 and an active network of 150 individuals [19], with each layer being inclusive of those within it. These layers represent natural disjunctions in both the level of intimacy between individuals and the frequency with which they interact, and are well established in the human literature [9,11,19,35,39–44]. It seems that these layers reflect constraints imposed by the fact that available social time is limited and so must be apportioned among relationships of different quality in such a way as to optimise the benefits they yield relative to the costs of maintaining relationships of the appropriate quality to provide those benefits [14–16,19].

We will focus on the two innermost (5 and 15) and the outermost (150) layers. Note that these layers are conventionally counted cumulatively [19]: successive layers consist of 5 strong relationships, 10 medium relationships and 135 weak relationships. For present purposes, we will overlook the intermediate 50 layer, since this would require us to add a further layer-specific functional benefit, and so add significantly to computational complexity on what is already an unavoidably complex model. We define any strategy that yields this pattern as being ‘structure-compliant’, meaning that it correctly matches this pattern. We ask three questions. First, given that multi-level structuring is rare in the natural world, are such social systems also rare in the model, with unstructured or small group patterns being more common (i.e. easier to evolve)? Second, what kinds of relationship strategies yield the layered distribution of exactly these sizes? Our third question is: under what fitness regimes does this multi-level structuring arise?

## Modelling the Emergence of Social Structure

The central assumption of our model is that different kinds of relationship provide different kinds of benefits [19], and that it is the balance of the trade-offs between these different benefits and the costs of servicing the relationships underpinning them that gives rise to structured social groups, with these benefits being maximised by living in groupings of different size [19]. In effect, multi-level social systems allow individuals to live simultaneously in the several different groupings that they need to maximise their fitness, whereas unstructured societies with a single optimal grouping size are likely to emerge when one fitness function overrides all others. For present purposes and for computational convenience, we will focus on just two kinds of benefit: defensive alliances (that provide close social or emotional support as well as protection against attack by conspecifics) and information exchange (essentially, foraging efficiency), both of which are deemed to be important for animals as well as humans [9,45,46]. However, we should emphasise that the specific benefits we use as our exemplars are less important than their functional characteristics: what is important is not the benefits *per se* but how they relate to relationship quality, the time costs of servicing them, and the impact they have on fitness. So long as there are at least two benefits that differ in the way we define below, our model is general.

The proximity of those who can be trusted to come to one’s aid should reduce stress by their reassuring presence and the implicit promise of intervention in conflicts, as has been documented in primates [20,21,47]. Among humans, there are striking effects of close friendships on wellbeing. Fowler and Christakis, for example, have shown how the happiness of friends and friends-of-friends can influence an individual’s happiness, and that this effect extends to various fitness-related conditions such as illness, obesity and even mortality risk [48–50]. Similarly, among baboons, females who are better embedded within their social networks live longer and have more surviving offspring [22–24].

Even though large support groups will always be more effective than smaller ones, size may be constrained for three reasons. First, only so many individuals may be able to provide the benefit at any one time (a too-many-cooks-spoil-the-broth effect). Research in the social psychology of group work confirms that benefits are asymptotic: larger groups commonly fail to perform in proportion to their size [51] for a number of reasons, including freeriding and loss of motivation [52]. Second, close relationships are usually reciprocal [44], so while each relationship accumulates potential benefit to an individual, it does so at the cost of exposing that individual to the risk of being called upon to reciprocate commitments to all of those from whom it receives the benefit. Third, the underlying basis of the trade-off is that, if the quality of a relationship (and hence its reliability) is a function of the time invested in it [11,19], there will be investment costs to creating and, in particular, maintaining such relationships. A cost/benefit trade-off will typically favour limiting investment in a small subset of individuals (‘special friends’ *sensu* [5]) rather than spreading one’s available social time budget more thinly among many individuals (see also [53]).

By the same token, the ecological benefits that derive from group-living (whatever these may be) will likewise be subject to diminishing returns as the costs incurred by living in ever larger groups increase. In this case, the trade-off will be due to the ecological costs incurred by maintaining a large group (mainly increased day journey lengths, direct foraging competition and/or disrupted time budgets: [54]) as well as the difficulty of investing enough time in these (weak tie) relationships to make them work and, importantly, to maintain group coordination [55].

Our model is thus based on two key assumptions: (i) Relationship strength is directly proportional to the frequency of social interactions, although the incremental increase in relationship strength is subject to a law of diminishing returns; and (ii) Relationship strength wanes over time. This core model is justified by the empirical observation that emotional closeness in relationships increases as a function of rate of interaction and then declines more gradually unless maintained by frequent social contact [11,56]. Sutcliffe and Wang [57] demonstrated that, in agents with different social preference strategies, the trust:interaction frequency model produces a law of diminishing returns only with a logarithmic increase and linear decrease function. This model of reciprocal social interaction leading to the emergence of social relationships was robust across a range of waning and reject (non-cooperative behaviour) rates. We here extend this analysis by using the same model to investigate whether structural layering composed of the inner intimacy layers (5,15) and the outermost layer emerge naturally with a wider range of objective functions, population size, agent strategies and initial conditions.

### Social Simulation Model

The simulation reused the Sutcliffe and Wang [57] trust model as the agent interaction/trust mechanism, together with the agent strategies introduced by Sutcliffe et al. [19] (for details, see S1 File, design is summarised in Fig B in S1 File).

We modelled the competing demands between time spent feeding (to maintain survival) and time spent socialising (to ensure the benefits of social cooperation). For computational simplicity, we restricted the model to a simple trade-off between social and foraging/feeding time. We used an agent-based model in which, on each round, agents faced the choice between socialising and foraging, and then, when socialising, which agent to interact with. Agents initially meet randomly, but remember whom they have met, so that in subsequent rounds they apply preferential strategies to constrain whom they socialise with. Each interaction was associated with two key process variables: a reject risk (the probability that a given interactee will not cooperate) and a waning rate (the decrease in relationship strength as a result of a previous failure to interact with a given agent). Interaction frequencies between all agent dyads were recorded as a relationship strength which was divided into terciles, allowing us to categorise relationships produced during each generation as strong, medium or weak. Strategies were modelled stochastically, so an agent who preferred interacting with upper tercile agents (i.e. strong relationships based on high interaction frequencies) might select relationships in the upper tercile on most, but not every, interaction. Each agent in the population interacted once in each cycle, and, after 2,000 cycles, fitness selection was applied to produce a new generation. In a typical simulation, the model was run for 50 fitness selection events (generations). There was no inheritance of relationships across generations. Details of the algorithm and variables are given in S1 File (Appendix).

In each run, we considered a population of 300 agents, equally divided between four different social strategies based on their preference for relationships of a particular strength. Relationship strength was defined as a function of the frequency of interaction, with four different social strategies defined in terms of this preference: *Favour-the-Few* (FtF) prefer to prioritise relationships in the upper tercile of the strength distribution, *Favour-the-Weak* (FtW) favour those in the lower tercile, and *Favour-the-Medium* (FtM) those in the middle tercile, while the fourth strategy (*Staged*) begins by adopting FtF but then progressively favours FtW as it builds relationships. An agent inherited its parent’s strategy (with breeding from the top 20% of agents ranked by fitness).

To investigate how well each strategy performed, we defined five criteria (or objective functions) that contributed to an agent’s overall fitness: three (wellbeing, alliance formation and resource acquisition) made positive contributions, while two (risk and stress) had negative effects and so functioned as costs additional to those incurred by the need to invest time in relationships. We can think of these are, respectively, the benefits and costs of forming social relationships. The detailed definitions are given in S1 File (Appendix), but broadly speaking wellbeing (WB) is a positive function of the average strength of an agent’s relationships, modulated by the attention it receives from others; the benefit from alliances (AL) is a positive function of the average strength of the agent’s relationships, modulated by the number of relationships it has; resource acquisition (RS) is a positive function of the number of foraging turns it takes; risk (R) indexes the agent’s risk exposure as a negative function of the number of social interactions it engages in (the more interactions, the higher the risk–that, for example, it will be called upon for active support when an ally is attacked); and stress (ST) is a negative function of the number of relationships the agent has [20], reflecting the beneficial effect that being in larger groups has. In different runs of the model, we varied the relative weightings of these five objective functions of a scale of low (1) to high (5).

We determined each agent’s fitness after a lifetime of 2,000 interaction cycles by, first, normalising its scores on each criterion by ranking them across the population, then modulating these ranks by the weightings selected for each simulation run, and finally summing these to create an aggregate fitness, defined as Fitness = (R + WB + AL)–(RS + ST). We then adopted the simple (but widely used) strategy of eliminating the weakest 20% of the population, ranked by fitness value, at the end of each round. Breeding replaced these by allowing individuals from the top 20% to replicate, thus maintaining population size at N = 300. Though high, the mortality rate we use is a reasonable approximation based on estimated death rates among hunter-gatherer communities [58]. Simulations were run for 50 generations to produce outputs showing the populations of agent strategies across generations, with average relationship strength divided into strong-, medium- and weak-tie ranges.

### Experimental Design

Our experimental design was intended to evaluate two questions: first, are there consistent patterns generated by the model in the total number of ties an agent has and their distribution between the different tie categories (strong, medium and weak) and, second, how do any such patterns map onto the fitness landscape (indexed by objective function settings). First a total of 3,125 simulations were run to investigate all permutations between the objective functions by systematically varying the weighting for each function from 1 to 5 (the minimum and maximum values) in integer steps so as to map the entire fitness landscape. Each run returned the frequency of ties, split by tie strength (weak/medium/strong) averaged for all the agents in the population (300). We then used cluster analysis [59] to aggregate runs by similarity in the number of the different categories of tie (strong, medium and weak). We used k-means analysis to determine the optimal separation of clusters; k-means clustering finds the best fit to the data by finding the k means that best describe the data (i.e. that minimise the variance). For present analyses, we varied k across the range 1–7 to find the optimal number of clusters. This allowed us to explore both the robusticity of the different patterns and identify the fitness criteria weightings required to yield each pattern. Next, we ran a second series of simulations in which alternative social strategies competed directly against each other, with a focus on the FtF strategy that had emerged as a key influence on social structure in the first series of simulations. Finally, we ran a series of sensitivity analyses to check whether there were confounds due to the use of a particular population size and selection regime.

## Results

### Model outcomes and their characteristics

**Fig 1:**
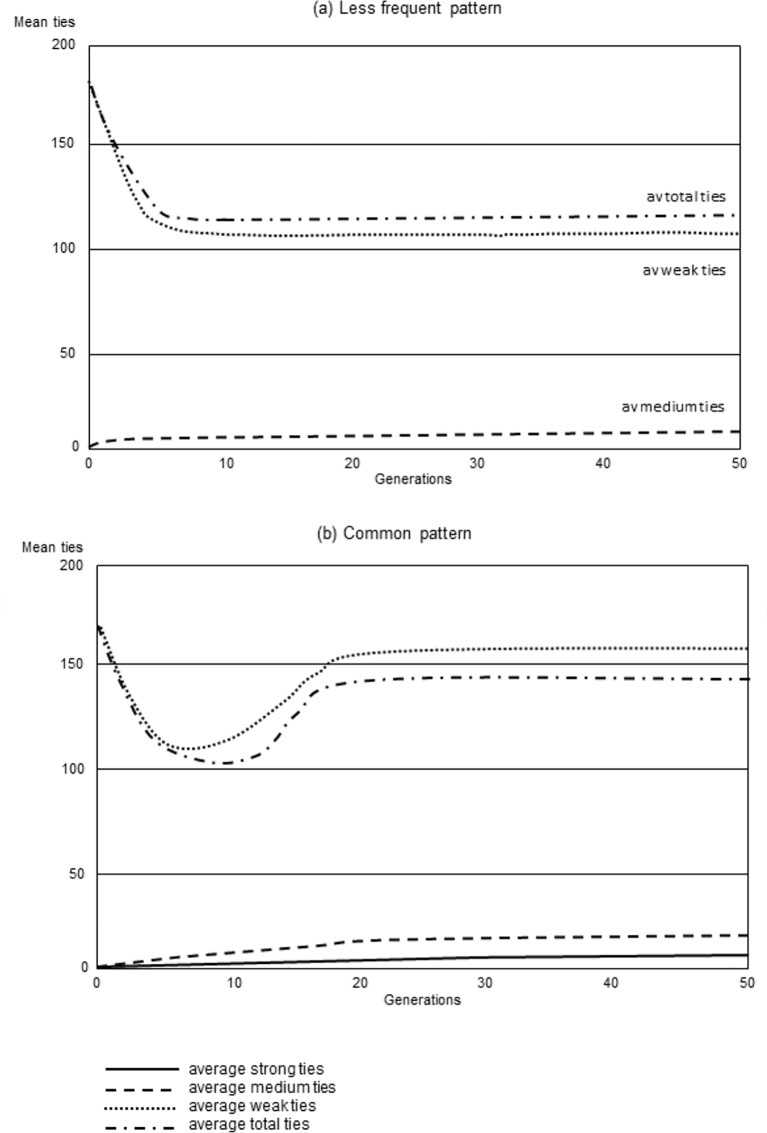
Sample results showing the mean frequency of ties/agent for the whole population over 50 generations for (a) one of the less frequent patterns and (b) the most common pattern. The plotted examples are the outputs at the end (generation 50) of two individual runs.

In the initial run, the parameters of the model were varied systematically across each objective function parameter space in order to determine the distribution of the various outcomes and the parameter weightings that characterise them. A two-level k-means clustering algorithm was used to find the optimal number of clusters with the smallest standard deviation for each cluster. Five clusters were identified as optimal (Table 1). These varied mainly in the frequencies of strong and medium ties and by total network size; only one of these sets mapped at all closely to the target distribution of tie types (i.e. were structure-compliant). Fig 1 illustrates the evolution of two of these (the dominant, or most common, pattern and the structure-compliant pattern) across generations in the simulation.

**Table 1:** Mean number of agents in each layer in the four different final social patterns identified by cluster analysis.

		Average agents (standard deviation)			
Pattern	Frequency	Strong	Medium	Weak	Total
Small core	2504	1.02(0.14)	6.91(0.40)	104.66(0.87)	112.59(0.99)
No-layers	437	0.02(0.23)	0.10(0.91)	120.61(1.03)	120.73(1.00)
Large core	155	7.90(0.59)	20.83(1.12)	134.29(1.80)	163.03(3.36)
Structure-compliant*	29	5.42(0.96)	15.98(1.84)	125.88(3.24	147.28(5.94)

* with layers of 5 strong, 15 medium, and 135 weak relationships, as found in humans.

The most frequent pattern (80% of total runs) was a ‘small core’ network with ~1 strong and ~7 medium ties, and an overall network of around 112 members. The second most frequent pattern (accounting for 14% of all runs) had no layers, and an average network size of ~120 members, nearly all of which were weak ties. We refer to these as the ‘small-core’ and ‘no-layers’ patterns. The third most frequent pattern (‘large-core’ pattern) had a large core with ~8 strong ties, ~21 medium ties and an overall network of ~160 individuals, but these were rare by comparison (5% of all runs). Finally, structure-compliant networks which mapped closest to the predictions of SBH [19,36] had an average of 5 strong ties, 9 medium ties and a network size of 147, but were extremely rare (1% of all simulations).

Table 2 maps the weightings for each strategy cluster on the five fitness criteria (resources, wellbeing, alliances, risk and stress). The small-core pattern had an even distribution of weightings in the range 2.87–3.16 for all five fitness criteria; while the no-layers pattern had similar resource and stress, it had much lower wellbeing and alliance (<2) and much higher risk (4.3). The function weightings of the structure-compliant and large-core patterns were generally similar to each other, with low resource and stress and high wellbeing and alliance.

**Table 2:** Mean fitness weightings for each social structure pattern in the first experiment with an initial equal distribution of agent strategies.

		Mean weighting (Standard deviation)				
Pattern	% total runs	Res	WB	AL	Risk	Stress
Small core	80.13	3.07(1.40)	3.16(1.38)	3.13(1.36)	2.87(1.37)	3.13(1.40)
No-layers	13.95	3.22(1.40)	1.79(0.93)	1.69(0.86)	4.31(0.85)	2.77(1.40)
Large core	4.96	1.52(0.72)	3.68(1.32)	4.41(0.80)	1.68(0.92)	1.68(0.88)
Structure-compliant	0.93	1.90(1.05)	3.72(1.25)	4.24(1.09)	1.83(0.97)	2.14(1.22)

Res = resources, WB = wellbeing, AL = alliance formation.

These results indicate that multi-level social structures of the kind found in primates, and especially humans (i.e. structure-compliant patterns), are extremely rare. The default pattern involves social systems with a relatively small number of weakly structured or undifferentiated relationships. Compliant and near-compliant patterns, by comparison, are extremely rare, accounting for barely 6% of all outcomes. The fact that there is significant clustering of outcomes indicates that structure-compliant patterns are not simply the extremes of a random distribution. Rather, they seem to occur only under a limited range of conditions (when the benefits of wellbeing and alliances is high, and the resource benefit is low). If these conditions do not hold, animals seemingly gain no fitness benefits from structure-compliant social organisations and are better off with looser, weakly structured social arrangements.

**Fig 2:**
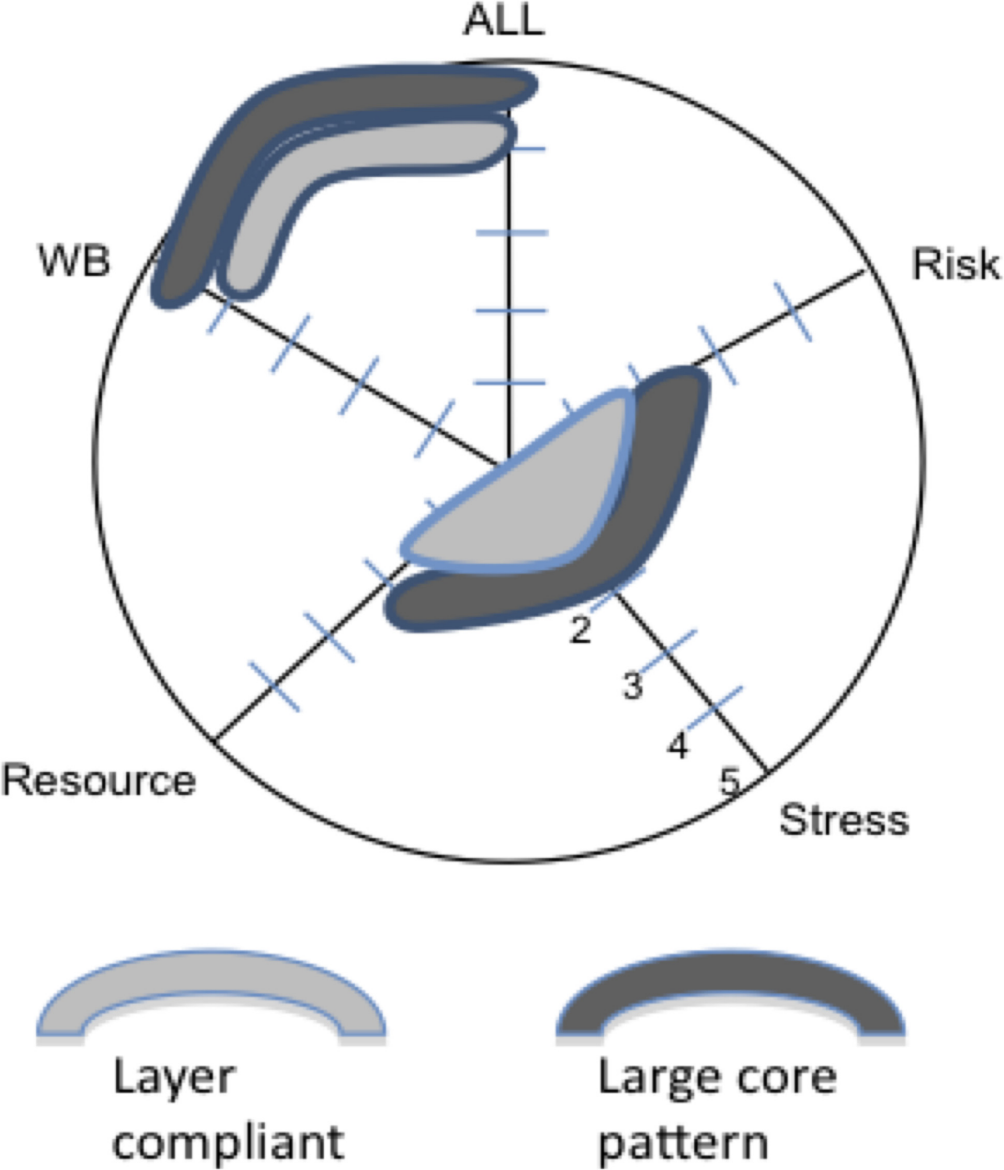
Locus of the layer-compliant and large core patterns in the space of fitness criteria weights. Dark shading indicates the layer-compliant pattern, lighter shading the many strong ties pattern. All = Alliance, WB = Wellbeing fitness criteria. Weights increase towards the circumference of the circle.

To explore this pattern further, cluster analysis was applied to those runs that produced a structure-compliant pattern in order to investigate possible groupings of the fitness criteria that give rise to this pattern. The average weights for the criteria in each cluster of the structure-compliant runs are shown in Table 3. In cluster 1, alliance and wellbeing as well as stress were high; cluster 2 showed high alliance with modest resources but low wellbeing; while cluster 3 had high wellbeing and alliance weights. Overall, a structure-compliant social structure emerged when either alliances or wellbeing contributed strongly to overall fitness, and the influence of risk and resources was moderate or low. The alliance criterion appears to be slightly more influential than wellbeing in determining structure-compliant patterns, showing a high average weighting in three of the four clusters. These results are summarised in Fig 2. Structure-compliant and large-core patterns occupied a relatively small part of the overall fitness space where the wellbeing and alliance criteria are favoured and other criteria have low weights. The rest of the space was occupied by the small-core and no-layer patterns.

**Table 3:** Average weights of fitness criteria for clusters producing the layered pattern found in humans.

Cluster	Resource	Wellbeing	Alliance	Risk	Stress	Percentage
1	1.0	3.0	4.6	1.0	3.9	15
2	2.4	1.8	4.3	1.4	1.0	25
3	1.6	4.3	4.8	2.5	2.0	30
4	1.6	4.8	3.1	1.1	1.3	30

### Competiveness of alternative social strategies

To investigate the competitiveness of the different social strategies, we ran further simulations with a population of 300 agents each characterised by three key traits, each with four variants:

- Social preference strategy: FtF vs FtM vs FtW preferences, plus a staged strategy as defined previously;
- Social time predisposition, indexed as forage:social ratio with four settings: 8:1, 5:1, 4:1, 1:1;

A cooperate:reject ratio with four settings, representing 1, 2.5, 5 and 7.5% of agents to simulate an increasing propensity to reject a social interaction.

Twenty simulations were run with a range of weightings for the fitness criteria that had produced either the small-core, structure-compliant or large-core patterns in the previous results, selected to sample the range of fitness settings reported in Table 2. The seed population (N = 300 agents) had a hierarchical structure, containing an equal proportion of agents by strategy and then within each strategy an equal number of individuals for each forage:social variant, and finally within each of these variants an equal number of individuals for each cooperate:reject variant. As before, the lowest 20% of the population was eliminated at the end of each round and the top 20% were allowed to reproduce.

Fifty simulations were run and the proportions of surviving agents after 50 generations are shown in Table 4. In the structure-compliant and the large- and small-core patterns, the staged and FtF strategies dominated, while FtM and FtW strategies dominated only in the no-layers pattern. The dominance of the FtF and staged strategies in simulations which produced structure-compliant patterns with few strong and medium ties, and many weak ties, suggests that the social strategy of favouring the few competes successfully even in the face of rejection: indeed, rejection decreased to nearly zero despite rejecting agents being rewarded with extra foraging turns and resources. The forage:social time budget ratio also changed to favour more social turns.

**Table 4:** Average (standard deviation) number of agents by strategy for each of the four social pattern outcomes in 50 simulations.

	Staged	FtF	FtM	FtW	FS
Small core	149.39 (1.91)	149.58 (0.97)	0.63 (0.02)	0.40 (0.1)	0.498 (0.07)
No-layers	2.18 (0.06)	1.62 (0.01)	273.62 (2.45)	22.58 (1.32)	0.499 (0.05)
Large core	138.38 (1.65)	161.55 (1.56)	0.03 (0.00)	0.05 (0.00)	0.152 (0.01)
Structure-compliant	134.34 (1.89)	165.66 (2.11)	0.00 (0.00)	0.00 (0.00)	0.266 (0.03)

FS = final forage:socialise ratio for each social pattern.

Forage:social ratios stabilised at 0.26 and 0.15 in the structure-compliant and large-core patterns respectively, whereas in the two more frequent small-core and no-layers patterns the ratio stabilised around 0.49, or an even ratio of turns. Strategies which formed stronger ties were favoured even when the criteria did not reward them (as in the fitness setting which produced the small-core pattern). However, selection on the forage:social criterion modified the survival of stronger relationships. The small-core pattern produced few stronger ties because each agent had proportionately fewer social turns compared with agents in structure-compliant populations; hence, the strong ties that did develop waned as a consequence of less frequent social interaction.

### Sensitivity analysis: initial conditions

**Fig 3:**
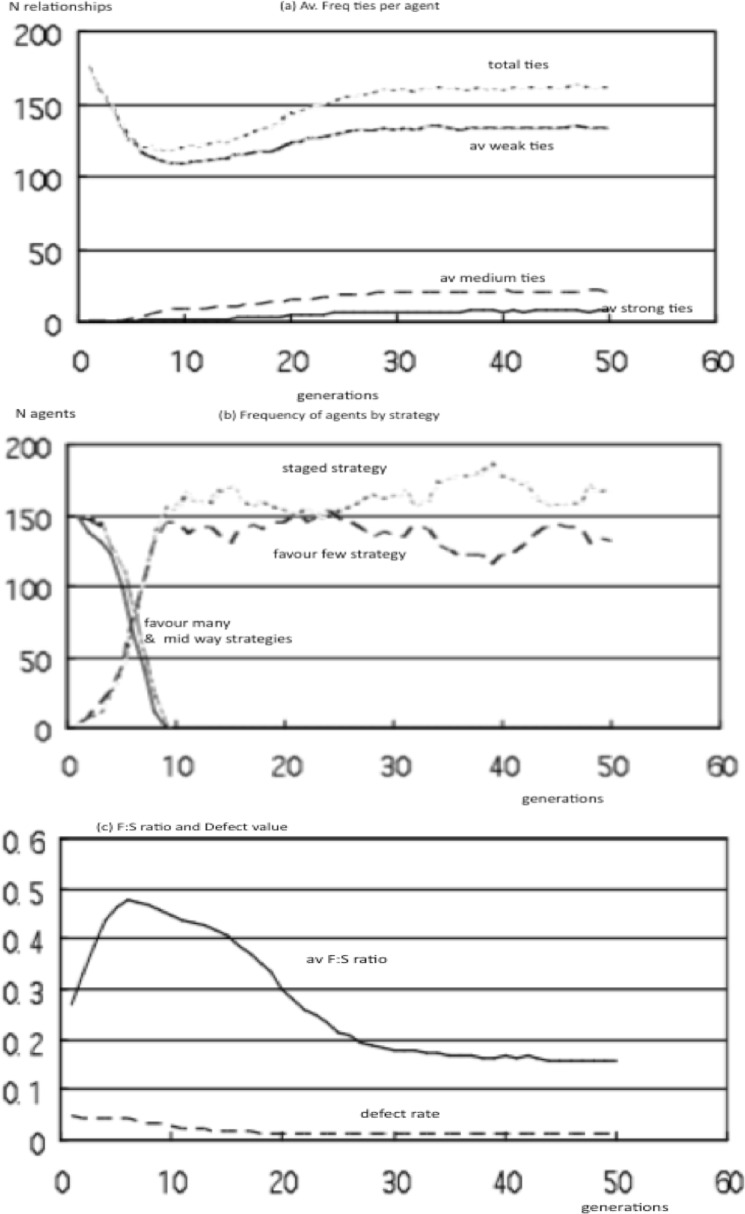
Model outputs with 1% seed populations of FtF and staged agents, (a) average ties/agent, (b) surviving agents by strategy, (c) Forage–Social ratio and defect rates for layer compliant pattern with weightings R:WB:AL:Risk:Stress of 1:5:4:1:2.

Since the FtF and staged strategies were competitive over a wide range of fitness criteria, we tested their ability to spread in populations (N = 200) initially dominated by FtW agents. When populations were seeded with 1% strong or 1% staged agents in a population of weak-tie agents with forage:social and cooperate:reject settings assigned at random using a roulette algorithm, both FtF and staged strategies spread rapidly to dominate the population within 10–20 generations. The model outputs (Fig 3) were the same as in populations that started with an equal distribution of strategies, with structure-compliant patterns being produced under a range of previously observed fitness criteria weightings. As before, the forage:social ratio stabilised at 0.25 while the cooperate:reject ratio was driven down towards the minimal setting with an average 0.05. The same result was produced with 0.5% seed populations of staged and FtF agents, and was robust across population size, so it appears these strategies have a strong competitive advantage over a wide range of fitness criteria.

We then tested the emergence of strategies and patterns when agents had no prior preference, i.e. their choice preference was randomised from all strong FtF to no favouritism. For N = 300 populations, two structure-compliant patterns emerged from the cluster analysis (Table 5). One cluster had the appropriate average number of strong ties (5.56) with slightly more medium ties than expected (18.94), while the second cluster, labelled ‘close to compliant’ had slightly more strong (6.92) and medium ties (19.21). (Note, however, that these results are both within the statistical variation of tie frequencies observed in human populations [19,35].) The best-fit run within the structure-compliant cluster had 4.32 strong ties, 15.74 (medium) and 127.65 weak ties, with objective function settings of Res = 1, WB = 1, All = 5, Risk = 4 and Stress = 2. The distribution of ties in the other cluster patterns (no-layers, small-core, large-core) were similar to those observed in the stratified initial population.

**Table 5:** Mean number of agents in each layer for the five different final social patterns identified by cluster analysis in the random initial condition.

	Average number of agents (standard deviation)			
Pattern	Strong	Medium	Weak	Total
Small-core	2.14(0.34)	13.84(0.70)	156.72(0.88)	172.71(1.99)
No-layers	0.00(0.03)	0.02(0.01)	186.66(2.03)	186.82(1.00)
Large-core	16.08(0.99)	31.1(1.16)	142.43(2.1)	185.59(3.36)
Close-to-compliant	6.62(1,2)	19.21(1.45)	133.14(1.89)	156.98(2.98)
Structure-compliant*	5.56(0.96)	18.94(1.84)	136.62(3.24)	161.14(2.94)

In the structure-compliant, close-to-compliant and large-core patterns, the majority of the surviving agents had parameter settings that matched the FtF strategy (93–99%), while the converse was true for the no-layers pattern (90% FtM). The structure-compliant pattern was considerably more frequent (38.6%) than in the previous stratified simulation (0.93%), while objective function settings for the cluster had higher resource, risk and stress but lower wellbeing and alliance weightings than in the stratified population simulation (see Table 6). The close-to-compliant cluster had similar average objective function weights as the structure-compliant result. The distribution of objective function weights in the other patterns was similar to the stratified population simulations, although values varied. Across the simulations, the forage:social ratio stabilised at a slightly higher value, 0.35, while the cooperate:reject ratio was driven down towards the minimal setting with an average 0.05.

**Table 6:** Mean fitness weightings for each social structure pattern in the random initial condition (N = 300 populations).

		Mean weighting				
Pattern	% total runs	Res	WB	AL	Risk	Stress
Small-core	12.7	3.16	3.11	2.34	3.73	2.92
No-layers	19.5	3.38	2.23	1.8	4.26	2.53
Large-core	20.0	2.15	3.63	4.05	1.93	2.38
Close-to-compliant	9.1	3.09	3.0	3.41	2.47	3.50
Structure-compliant	38.6	3.15	3.02	3.17	2.79	3.46

Res = resources, WB = wellbeing, AL = alliance formation.

In sum, the agent parameter settings were similar to those previously obtained, indicating that our findings are robust to variation in initial conditions. More importantly, it appears that, under the appropriate conditions, the FtF strategy is competitive and produces structure-compliant patterns across a range of objective functions; indeed, if anything, a random initial setting is more favourable for the emergence of structure-compliant patterns.

### Sensitivity analysis: population size and selection rate

When we varied population size in the previous section’s simulations, we used the same process mechanism values (trust-formation smoothing ratio and waning rate) for all populations, irrespective of size. For a smaller population, N = 200, the results for emergence of the FtF strategy and compliant patterns were similar. Since a larger population will result in a decreased frequency of inter-agent interaction, we ran a further set of simulations with populations of N = 500, increasing the strength of the trust-formation smoothing ratio (CR) to 20 and a proportional increase in the waning rate to 0.18. As before, the results were dominated by the no-layers and small-core patterns. However, in contrast to the N = 300 populations, the larger population simulations did not produce any structure-compliant or large-core patterns. This result was also observed for the random initial population. Since the decreased probability of socialising may have reduced the persistence of strong relationships, additional simulations were run extending the parameter range for the fitness criteria from <1…5> to <1…10>. Increasing the fitness criteria range produced structure-compliant and large-core patterns with similar clusters to those observed in the N = 300 population, but with increased wellbeing and alliance weights at 7–8, and low resource, risk and stress weights. The random agent preference condition produced similar results but with a lower proportion of structure-compliant runs.

This suggests that the model outcome scales to population size, given appropriate adjustment of parameters to reflect the fact that less frequent interactions are inevitable within larger populations. However, a population of 500 individuals is, even by human standards, large for an individual to have any kind of regular contact with or knowledge of, and the fact that structuring does not evolve so easily in larger populations has important implications for the evolution of sociality within the human lineage. It seems that when large populations occur, structuring can occur only when either the trust intensity or the fitness benefits, or both, are proportionately higher, reinforcing the initial conclusion that multi-level structuring occurs only within quite a limited range in the parameter state space.

Since selection at 20% represents a severe ‘mortality’ rate, simulations were also run with selection rates of 5% and 10%. These reduced the number of structure-compliant patterns in the same fitness space, with only one structure-compliant result being produced at 10% mortality (weightings of 2-3-5-1-3 for RS/WB/AL/R/ST, respectively) and none at all at 5%. Instead, populations were dominated by no-layers and small-core patterns with a forage:social ratio close to 0.50. High mortality thus seems to be an important precondition for the evolution of structure-compliant social systems.

In sum, between them these simulations suggest that emergence of structure-compliant social structures, such as those found in many primates and in humans, are influenced by both the selection rate and population size, with modest population size, high mortality and skewed reproduction being especially critical.

## Discussion

Within the assumptions of our model, selection favours two patterns in a wide variety of environmental contexts. The most frequent, the small-core pattern, is produced by the persistence of strong ties and staged-strategy agents with the tendency to FtF appearing in nearly half (45%) of the simulations. Such a pattern might describe the kinds of social system found among species that live in monogamous pairs or small cohesive groups (e.g. the social hunters and many of the harem-forming species among the ungulates and New World primates). The second most frequent pattern (no-layers, perhaps the category into which most herd-forming mammals fall) is driven by a mixed population of agents with medium and weak tie social preferences. The first thus favours small groups (pairs or small harems), while the second favours weak-tie social goupings without layers (e.g. large diffuse aggregations).

By contrast, a structure-compliant (i.e. multi-level) pattern–the pattern peculiar to a relatively small number of mammal taxa–emerges only under a limited range of conditions when the benefits of alliances and wellbeing are high, and resource competition is low. The first two patterns show a strong tendency to increase foraging time at the expense of social interaction, with the forage:social ratio migrating towards 1:1, whereas the last shows a reverse tendency to favour social interaction over foraging. There seem to be two conflicting trends here, one promoting stronger ties by FtF in social interactions with less foraging (compliant, large core), while the other favours foraging time at the expense of social interaction and a smaller social core (small core, many weak ties).

This polarisation reflects the competing rewards that arise from maximising individual fitness by devoting more time to foraging versus those that derive from improved survival accruing via social benefits. Exposure to high levels of risk and/or stress appears to prevent stronger ties from forming. The weak ties pattern therefore dominates for most fitness criteria combinations, i.e. for all runs except where wellbeing and alliance are high (4–5) and other fitness criteria are low (1–2), suggesting that social structures with many weak ties will emerge under a wide range of fitness conditions–as seems to be the case among birds and mammals generally.

The structure compliant pattern was more common in populations with an initial random distribution in social preference, and infrequent when initial agent populations had separate strategies. Mutation and selection on the continuous strategy population appears to promote survivorship of agents biased towards FtF. This suggests that, within a gene pool with an initial random social bias, evolution will favour the FtF allele and structure compliant patterns, whereas when other social strategy biases (FtM, FtW) are present in the initial population these may provide more effective competition. A few FtF genotypes also spread in majority FtW populations, so this allele appears to have selective advantage over a wide range of OF conditions. Although our model does not account for other possible explanations for the emergence of social structure, e.g. power gender asymmetry in harem groups, we note that a parsimonious model based on the single social affinity tendency we modelled did account for the emergence of a range of social structures including harem groups (small core pattern).

Theoretical models of social behaviour among unrelated individuals have focused on the evolution of cooperative behaviour and altruism [59–61], where reciprocal rewards accruing from social relationships outweigh the costs of social interaction, thereby providing the incentive to invest in cooperation. Cooperative behaviour spreads rapidly when there is a pay-off from repeated encounters with individuals within a population, as demonstrated in many models based on the repeated prisoner’s dilemma (RPD) paradigm [62]. Cooperative strategies with strong reciprocity compete effectively with non-social defectors in social dilemma games across a wide range of models and frameworks [63], so it appears that reciprocity, which is a key attribute of trust in human social relationships [64], underpins cooperative behaviour. While cooperation spreads when there is memory of previous encounters with specific individuals, it can also spread when memory of an individual’s reputation for previous cooperative acts is visible as an ‘image score’ [59]. Reciprocity in our model was reflected in social strategies (in particular FtF) which progressively restrict social interaction to a few favoured individuals; strong ties therefore encouraged reciprocal responses. The effect of trust in promoting the development of social relationships was demonstrated by Nowak and Sigmund’s [59,60] studies on reputation (‘image scoring’) in a coalitional version of the prisoner’s dilemma game: they showed that cooperation and altruism are likely to be widely adopted in populations where reputations are publicly visible. Hardy and van Vugt [65] also proposed that reputation systems are a necessary prerequisite of evolutionarily stable cooperation in large groups. Furthermore, Roberts and Renwick [61] demonstrated, in both experimental studies and computer simulations, that individual reputation based on histories of collaboration leads to the formation of social relationships.

These models may well explain patterns of cooperation among animals in general, but they do not of themselves predict the evolution of structured populations. Yet these are a class of societies that, while rare, are nonetheless conspicuous in some orders of mammals. Our model suggests the structure-compliant and the large-core patterns will indeed be rare, appearing only in a small fraction of the settings (8%) in the overall fitness space, implying that they evolve only under very specific conditions, namely when social rewards are high (notably in terms of wellbeing and the value of alliances) and social costs (risk and stress) are low. If social interaction with many individuals is not beneficial for agent survival, the Favour-the-Few (FtF) social strategy may still be competitive, but it gives rise to small group societies such as pairbonded monogamy or harem-like groups (the small-core pattern). Our model is a strictly functional model. This does not, of course, exclude a variety of other relevant explanations for particular relationships, including social forces such as romantic love and power asymmetry as explanations of pair bonds and harem relationships. However, Tinbergen’s Four Why’s [66] remind us that it is important to keep explanations of different logical status strictly separated, since they are complementary rather than competing explanations. Explanations in terms of romantic love and power asymmetries are mechanism-level explanations, whereas our model offers a functional level explanation that will naturally be exemplified in some kind of mechanistic process. Our model is concerned with the organisation of relationships (the emergent effect of their number, quality and structure), not with the physiological or psychological mechanisms that underpin these relationships.

Wittemyer et al.’s [67] elephant study shares with our model the assumption that time spent in social interaction influences relationship strength, but they adapted Heider’s [68] balance theory’s constraint that having too many connected friends increases the risk of conflict. As a result, in their model a few intense relationships emerged at the expense of more weak ties–albeit with optima similar to our findings (N = 5 strong ties). Our model also demonstrates that social preferences (i.e. FtF) can emerge when the benefits of alliances balance the risks inherent in cooperation, but adds the additional finding that further layers can emerge when other functions are being optimised at the same time.

Our findings also extend previous models by demonstrating that social network structures emerge when there is a preference for trustworthy individuals (i.e. ‘cliquers’: [69]). Hruschka and Henrich [69] used a modelling approach to show that social structures and preferential social relationships emerge when agents possess strategies favouring known collaborators. In these cases, social preference strategies (‘cliquers’) spread through populations across a range of cost/benefit ratios and defect rates. However, their model depended on a memory constraint, so only a limited number (10) of stronger relationships could be developed. Our model suggests that in the absence of such a memory constraint (or, rather, where memory capacity is greater than they assume), more complex structures can emerge.

The FtF social strategy emerged in different evolutionary contexts, although social interaction time being favoured over foraging was a common factor whenever this happened. This indicates that structured sociality emerges when the benefits of social interaction are more intense, and the impact of foraging time on survival is less critical. Such a pattern may be exemplified by the contrast between the relatively asocial folivorous monkeys that live in small social groups (N < 15) who are obliged to have a high feed:social time ratio by the demands of leaf fermentation (e.g. the New World alouattines and Old World colobines) and the more intensely social frugivorous monkeys that live in larger groups (N > 20) and can afford to have a low feed:social ratio (e.g. the cercopithecines) [26].

The emergence of stratified social structures that are structure-compliant is influenced by both the selection rate and population size, with high mortality (from all sources), skewed reproduction and small population size (or low density) being critical. This fits well with the fact that, at least among primates, predation risk increases as species become more terrestrial in habit [27,28,68] with the extreme case on both counts being represented by humans. This increase in risk of mortality maps well on to species’ differences in social complexity, in particular the extent to which they have structured (i.e. layered) social systems. Primates also suffer, uniquely, from unusually high risks of infanticide [70–72], which is likely to significantly exacerbate the impact of the mortality factor, and may help to account for the otherwise unexplained fact that structured social systems are unusually common in this order.

The clustering of fitness criteria observed for structure-compliant simulation runs gives support to our hypothesis that relationships at different levels of intimacy could emerge through different cost/benefit trade-offs. The two clusters that emerged had high alliance with relatively low wellbeing balanced against high stress in one cluster and a mix of stress and risk in a second. This pattern was also observed, albeit with less strong wellbeing and higher stress and risk levels, for the two structure-compliant clusters in the random agent condition. The details as to which particular values are critical for these parameters is not, of course, the issue here: the point is that mortality levels simply need to be *relatively* high compared to the norms for a given taxonomic group.

The emergence of social structure appears to be especially sensitive to both population size and the intensity of selection. Increasing population size makes the emergence and persistence of strong social relationships more difficult. Forming strongly structured social networks from within large herd-like groupings may therefore be difficult to achieve, which accords with the trajectory for the emergence of social structure from smaller rather than larger groups in most intensely social species (primates: [73]). Relatively high mortality or selection rates also seem to be important for the emergence of structured populations. Some of this will be due to predation risk (note that this reflects the risk of predation *before* the animals have managed to effect any anti-predator strategies, and not to the residual *rate* of predation: see [74]), but the risk of intra-species mortality (e.g. fighting) as well as infanticide [75] and simple failure to reproduce due to poor competitive ability (and low rank: [20,76–78]) will be contributory. Under such circumstances, effective social alliances may be an important component in overall survival, buffering the individual against both external sources of predation and internal sources of conflict. In such cases, multi-level structuring will emerge so long as the costs are not too high.

In short, the evolution of structure may critically hinge on whether it is foraging or predation-driven mortality that has the more intrusive influence on fitness, since the first will often be solved through individual trial-and-error learning whereas the second commonly requires social cooperation (especially in terrestrial diurnal species: see [27]). With increased cooperation, social species may be able to reduce their dependency on foraging time, thereby favouring an increased social time budget and hence higher frequencies of social interaction. Which trend bootstraps which, however, remains an open question.

In sum, the fact that population size and high mortality levels may be critical for the evolution of highly structured societies may explain (a) why these are relatively rare among mammals and (b) why they are especially characteristic only of primates (including humans). Indeed, most of those species of non-primate mammals that have multi-level social systems also seem live in predator-risky, terrestrial (or oceanic), open-country habitats.

## Supporting Information

S1 File Appendix. (DOCX)
