# Supplementary material for: Modelling the Evolution of Social Structure
Source: PLoS One. 2016 Jul 18;11(7):e0158605. doi: 10.1371/journal.pone.0158605 (PMC4948869; doi:10.1371/journal.pone.0158605)
Supplement: S1 File — (DOCX) [file pone.0158605.s001.docx]

**Appendix**

The model we use is an agent based computational model. Since the model involves a large number of parameters, we first outline in general terms how the model works and define the key parameters. The detailed dynamics of the model, and the equations involved, are given in the sections that follow.

**Model Overview**

The model simulates relationship development arising from cooperative interaction with a trust reputation mechanism. The key components are:

- Agents interact, thereby forming relationships that facilitate the acquisition of some fitness-relevant benefit. Trust, which is calculated from the frequency of interaction is equated with strength of relationship.
- Responding agents may choose to accept or reject an invitation to interact, hence relationship strength accumulates for positive interactions, and decreases with negative interactions.
- Each agent within the population is assigned a turn, so there is an equal opportunity to interact. Initially, the individual with whom an agent interacts is selected at random. However, once a history of interaction has developed, an agent’s choice is governed by its strategy.
- Agents have preferred social strategies that predispose them to favour interactions with specific individuals according to the history of previously successful interactions.
- Agents choose between devoting time (rounds in the model) to foraging or to social interaction. Each activity has different consequences for the agent’s fitness.
- An agent’s fitness is measured in terms of five core parameters, three of which positively influence fitness while two do so negatively.
- After a specified number of rounds (defining a generation), the top 20% of agents defined in terms of their individual fitness are allowed to reproduce, and the bottom 20% die. This allows population size to remain constant, while its composition evolves.

**Interaction and Trust Model**

The main components of the model are:

- Agents who interact and form trusting relationships. Frequency of interaction is equated with strength of relationships and trust.
- Responding agents may choose to accept or reject an initiation, hence interactions may have positive or negative results. Rejections, modelled as reject ratio have no benefit or social cost apart from the lost opportunity of relationship building.
- Trust accumulates for positive interactions, and is decreased by negative interactions.
- Agents have a memory of previous interactions with other agents.
- Agents have preference strategies for initiating social interaction according to the history of previous successful interaction.
- Increase in trust is controlled by smoothing algorithms to implement theoretical assertions that high-trust relationships exhibit different behaviour from low-trust relationships.
- Trust in relationships wanes over time at a slow rate independent of any interactions.

Each agent within the population is assigned a turn, so there is an equal opportunity to interact. Initially the other agent is selected at random. However, once a history of interactions develops, the focal agent’s choice of the other agent (or alter) is governed by a history-dependent response algorithm which selectively initiates interaction according to the agents’ strategy.

Trust accumulates from successive interactions between any two agents. The trust value is incremented by one unit for each cooperative interaction between A and B. Trust is an attribute of the relationship, which is a direct arc (or tie) between any two agent nodes (A🡪B, B🡪A), so reciprocal trust can be modelled. Initially trust increases linearly in proportion to the number of cooperative interactions between any two agents. However, to model the empirical observation that trust reaches an asymptote in human relationships, a law of diminishing returns is implemented so that, as trust in a relationship increases, a log ratio algorithm is applied such that the rate of increase progressively decreases as the value of trust rises. Since the log algorithm applies to negative as well as positive interaction, high-trust relationships are relatively immune to rejections. This models the intuition that people forgive alters’ occasional indiscretions in high-trust relationships.

The formula for log trust increase (large increase when the trust value is small, and small increase when the trust value is large), uses four parameters:

*Max_trust*

*Max_compression_ratio* (*MaxCR*)

*Min_compression_ratio* (*MinCR*) and

*Compression_interval* (*CI*).

*MaxCR* is the maximum trust increase when the trust value equals zero and *MinCR* is the minimum trust increase when the trust value equals *max_trust*. C*ompression_interval* (*CI*) is the range on the trust scale used to modify trust increase. The *compression_interval* is defined as:

(1)

Based on Equation (1), the trust increase at time (*t*+1) is defined as:

(2)

and,

(3)

From Equation (2), we see the increase of trust decreases with the value of current trust. By doing this, we realise a log trust increase.

In our experiments, we set *MaxCR*=10*, MinCR*=0, and *MaxTrust*=100. This produces:

according to Equation (1).

Applying Equations (2) and (3), we have

(4)

From Equation (4), we see, if , then and ; if trust at time *t* has the maximum value, then , and .

**Trust waning**

All relationships are assumed to require periodic maintenance but strong ties tend to persist longer with less reinforcing social interaction. Waning is inversely proportional to the strength of trust; thus, relationships with high strength will exhibit less waning than relationships with low strength. To ensure that all trust relationships exhibit some degree of waning, the waning equation incorporates a minimal decrease even for strong trust relationships:

The log trust waning algorithm has two parameters, min_waning (*MinWaning*) and waning_rate (*WaningR*), which define the of Min_waning_cost as follows:

(5)

The formula for log waning is defined as:

(6.a)

Equation (6.a) can be simplified to Equation (6.b):

(6.b)

Trust can not be a negative value, so we modify Equation (6.b) to (7)

(7)

**Agent strategies**

A probabilistic *search function* determines how the focal agent chooses a target agent for interaction according to the history of previous cooperative interactions. Four strategies were implemented to model different preferences in relationships formation; three are static with a constant bias is alter selection, while the fourth strategy changes during the simulation run.

Agents are assigned one of four alternative social strategies:

1. *Favour-the-few (FtF)*: the history-dependent search function favours cooperating with individuals with whom the agent has had more previous successful interactions. This encourages development of strong ties. However, since the function is stochastic, there also is a low probability of initiating interaction with low-trust individuals and/or strangers without a previous interaction history. As relationships develop, the search function becomes progressively biased towards existing stronger ties (i.e. agents prefer to interact with those with whom they already have the strongest ties).
2. *Favour-Medium-Ties (FtM)*: the search function is biased towards the mid-point in the trust relationship distribution (i.e. agents prefer to interact with those with whom they have ties in the middle of the distribution).
3. *Favour-the-Weak (FtW)*: search is biased towards the low trust/interaction frequency part of the distribution; agents thus favour new and unfamiliar ties.
4. *Staged*: in this dynamic strategy, the search bias changes with time such that the agent initially favours strong ties but progressively favours initiating proportionately more interactions with strangers and ‘weak tie’ partners. This implements the behavioural predisposition, manifest over an individual’s lifetime, that strong ties are formed earlier in life, while weaker ties accumulate in later life (Hays 1989).

In the random conditions, agents’ favouritism is randomly distributed in a vector (0..1) which biases their behaviour from zero through a range across FtW to FtF.

**Interaction Process and Relationship Formation.**

We consider the *i*th agent as the focal ego. The probability for the focal ego (ego *i*) meeting a new agent is

, (8)

where *N_t,i_* is the number of ties at time *t* for the focal *ego* (ego *i*), and *P* the number of agents in the whole population.

The probability of interacting with existing ties is

. (9)

Based on formulae (9), the actual probability of the focal ego (*i*) selecting an existing alter agent (*j*) is , where *p_ij_* is calculated according to the agent’s strategy (a-d below).

The function for all strategies first sorts all *alter*s having ties with the focal ego (ego *i*) by trust in descending order. Total trust for the focal ego (ego *i*) is the sum of trust in its ties with other agents:

, (10)

where *t_i,j_* is the trust of the focal ego (ego *i*) with the *alter* agent with trust rank *j* and *Nt_,i_* is the number of ties (egos have ties with the focal ego) at time *t*. Then,

- 1. The probability of agent *i* choosing to interact with agent *j* under the FtF strategy (strong ties preferred) for an alter with trust rank *j* is: .
  2. The probability of agent *i* choosing to interact with agent *j* under the FtW strategy (weak ties preferred) for an alter with trust rank *j* is: , where the index .
  3. The corresponding probability for the FtM strategy (medium ties preferred) for an alter with trust rank *j* is if the alter is in the top half of the ranking (based on descending trust ordering), or if the alter ego is on the bottom half part of the ranking
  4. The probability for the staged strategy to interact with an alter of rank *j* is: , where the cycle count, (with index ), *RC* is the number of remaining cycles in the life time and *cc* modifies the probability of selecting alter *j* through RC cycles.

We illustrate how alter selection strategies work in Figure A1. Given a focal ego with 5 ties, The trust for the alter relationships is ranked in descending trust order and *Nt_,i_* = 5, .

Figure A (a) shows strong ties preferred strategy; the probability of selecting an alter is proportional to the relationship trust to total trust in all the ego’s relationships. Figure A (b) shows the weak ties strategy; where the probability of selecting an alter is proportional to trust value in the inverse rank order so if *j* = 2, *Nt_,i_* - *j* + 1 – 5 = 4; if *j* = 3, *Nt_,i_* - *j* + 1 – 5 = 3; and so on. The medium tie strategy applies weak ties strategy for the top 50% of alters (ranked by trust in descending order) and applies strong ties strategy for the bottom 50% alters.

| selection probability |
| --- |
| *p_ij_*=50/150 |
| *p_ij_*=40/150 |
| *p_ij_*=30/150 |
| *p_ij_*=20/150 |
| *p_ij_*=10/150 |

| selection probability |
| --- |
| *p_ij_*=50/150 |
| *p_ij_*=40/150 |
| *p_ij_*=30/150 |
| *p_ij_*=20/150 |
| *p_ij_*=10/150 |

| rank | trust |
| --- | --- |
| 1 | 50 |
| 2 | 40 |
| 3 | 30 |
| 4 | 20 |
| 5 | 10 |

| rank | trust |
| --- | --- |
| 1 | 50 |
| 2 | 40 |
| 3 | 30 |
| 4 | 20 |
| 5 | 10 |

(a) FtF strong tie preferred strategy (b) FtW weak tie preferred strategy

**Fig A. Ranking for strong and weak tie Alter selection strategies**

The stage strategy works by applying the strong ties strategy in early cycles (in a model run) and then progressively applies a weak ties strategy in later cycles. So applying a strong strategy with 3000 cycles in a run, at the beginning when there are 3000 cycles left (RC) the probability for selecting the first ranked alter is:

and .

The second term for the right hand side is a very small value (0.8 x 10 ^-7)^, so initially the probability of selecting this alter for stage strategy approximates to the probability of selecting this alter with a strong ties strategy (0.33). If we are approaching the end of a model run, when only one cycle remains,

then and .

Hence at the end of run, the probability for selecting the top rank alter is the same as the weak ties strategy (0.067). If we are in the middle of the run, with 1500 cycles remaining then

and ,

which is smaller than the probability early in the run and larger than the probability at the and of the run.

**Model Control Process**

The top level algorithm for the model has two cycles. The first powers the simulation for the desired set of runs. The second cycle gives each focal agent a turn within a run and iterates according to the number of agents in the population.

Model-Control

Initialise-model (N agents, strategies, run-cycles, parameters CR, W, D, FS)

While Run-cycles Do

For Agent (i) to N Do

Select Focal Agent (i) {initially at random)

If Forage-turn Then

Increment Res by 1 {1}

Else {Social-turn}

Read Relationship History

Search for Alter-agent {2}

If Alter-response = Cooperate Then

Increment Trust by (MaxCR-CI x Trust) {3}

Else {Alter rejects}

Decrement Trust by (MaxCR-CI x Trust)

Increment Reject by 1

End-If

Decrement Trust by W {4}

End-Do

End-While

Notes

1. The forage: social turn is set by the FS ratio parameter.
2. Search depends on the agent strategy as described previously, so initially when there is no relationships history the search algorithm for all strategies selects a stranger at random. As relationship histories accrue the algorithm is chosen more selectively according to the agent’s strategy.
3. Trust compression is applied progressively as the relationship (i,j) increases. Initially there is no effect since there is no history.
4. Waning is applied as described above, with the adjustment in equation 7 so the value does not become negative.
5. The parameters Compression Rate for Trust, W for the Waning rate and Rej for % Rejection rate on response can be changed between experimental runs.
6. Trust defines the strength of the relationships between agents

More details of the algorithms and source code can be found at http://www.openabm.org/. searching using keyword ‘trust’ finds the model titled SBH trust model

**Objective Functions**

Each agent was characterised by five functional criteria that influence its fitness, defined as follows:

Wellbeing (WB) defined as: WB = mR *(I_in_/I_out_)

where mR is the mean strength of Trust in an individual agent’s relationships (defined by the frequency of interaction, such that mR=ΣTrust _i..j_/N averaged across all active ties; I_in_ is the total frequency of interactions initiated by other individuals with the agent during a lifetime; I_out_ is the total frequency of interactions initiated by the agent with other individuals during a lifetime. Hence, wellbeing is modulated by the balance of interactions, with individuals receiving more attention from others having better wellbeing than net donors of interaction.

Resource gain (R) is: R = F_r_ * N

where F_r_ is the total frequency of foraging turns by the agent in a lifetime, and N is the total number of the agent’s relationships at the end of its lifetime. We assume that food resource benefits accumulate as a function of the number of ties an agent has because such relationships provide information that improves an agent’s ability to find food. More ties (of whatever strength) provide more foraging benefit.

Alliance benefit (AL) is: AL = mR * (a* N)

where *a* is a coefficient that moderates the influence of total ties, set to *a*=0.05. Agents with more relationships and higher trust are favoured, although the tie volume effect is reduced by the coefficient to reflect the importance of stronger relationships. The coefficient models the relatively small number of relationships (a*N assuming N=150 relationships = 7.5 stronger ties) which are available to provide alliance support. This factor modulates the mean tie strength, such that stronger tie strength tends to provide more support in alliances.

Risk (RS): RS = mR / (b* I_tot_)

where *b* is a coefficient that moderates the influence of total interactions, *I_tot_* (where *I_tot_=I_in_+I_out_*); and *b*=0.005. Risk indexes the agent’s exposure to the need to reciprocate in the future, in a context where having to interact incurs costs: the more interactions an agent has, the more risk it incurs. The coefficient is set at 0.5% since risk is actually infrequent in all social interactions. Helping an ally, and hence risk exposure, is only necessary in extremis.

Stress (ST): ST = N

Stress is a simple function of the number of relationships that an individual has and reflects generic costs of living in larger communities. In most cases, these are reflected in physiological costs that, among other things, reduce female fertility (Dunbar, 1980, 1988; Abbot et al., 1986; Hill et al., 2000). Stress is present in all interactions even though the vast majority are friendly. Stress is a function of living in social groups and larger groups with more relationships induce greater stress. In practical terms, stress is simply the accumulation of the many casual incidents (displacements, ecological competition) that arise from living in close spatial proximity.

**Experimental design**

The experiment and model simulation process is summarised in Fig B. The model is run for a set number of cycles. This outputs ties for each agent with varying strengths. The average tie strength for all agents in the simulation is calculated and reported in a tercile split (Strong, Medium, Weak). At the end of a simulation run of N cycles, fitness is calculated using the Objective Function formulae. Fitness broadly depends on the number of relationships. The worst 20% of the agents ranked by fitness are eliminated. The best 20 % agents ranked by fitness replicate. Each agent in the top 20% has a random chance for replication. The model then runs N cycles for the second generation, and so on.


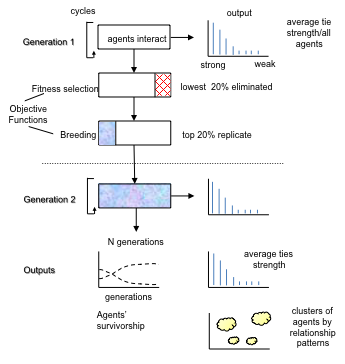


Fig B. Summary of model simulation stages and outputs

After the requisite number of generations have been run, the simulation reports the survivorship of agents by strategy over the generations, and the average tie strength of agents in the final population.

The experimental parameters (independent variables) in the model are:

- Reject rate: percentage of turns in which the respondent will not cooperate.
- Waning rate: a parameter that decreases relationship strength each cycle.
- Number of cycles (generations) in each model run.
- Initial proportion of agents by strategy in the population.

The outcome (dependent variable) is the strength of a tie between two specific agents, defined by the frequency with which they interact. Since the frequency of relationships by strength for each agent has a power law or J-curve distribution, a tercile split produced a few agents in the upper third of the range of strength, a few more in the mid-range, while most agents fell within the tail of a power law distribution. Relationships were summarised in discrete categories with strong ties in the upper tercile (33.3% of the range of strength) of the distribution, weak ties in the lower tercile, with medium ties falling between strong and weak.

Initial parameter values for the model were set at:

- Frequency-intensity algorithm: log increase/linear decrease
- Smoothing interval on increase = 10%: the log algorithm is applied progressively on each 10% of the range of relationship strength values (0-100)
- Waning rate = 0.08: strength of a relationship is decreased by 8% per cycle
- Agent social strategies: frequency set at equality for each strategy or a random preference distribution.
- Population size N=300.

**Summary of Parameters**

| Parameter | Description | Experimental settings |
| --- | --- | --- |
| T Trust (agent i- agent j) | Sum of previous interactions between agents i & j | Determined by interactions. |
| CR | Compression ratio (Min-Max) modulates change in trust/interaction | Min =0, Max 10 |
| CI | Compression interval | Trust increment determine by CR equation (4) |
| Rej Reject rate | Proportion of interactions in which the responding agent reject, hence the relationship strength is not incremented (no trust) | 0 – 10% |
| W Waning rate | Parameter controlling the decrease in relationship strength per cycle | 0.08 |
| Smoothing Interval | Parameter controlling the rate of trust increase /turn depending on the existing trust between agent I and j. See also CI. | 10% |
| Cycles | Number of cycles (interactions between all agents) per generation | 2000 |
| Generations | Number of interaction cycles each one followed by selection and breeding within one simulation | 50 |
| N Agent Population | Number of agents in the simulation | 300, some simulations with 200,500 |
| Agent strategies | FtF, FtM, FtW favour the few (strong ties), medium ties, weak ties, staged- see also Appendix A. | Initial population 33-33-34, FtF, FtM, FtW or random preference. |
| WB: Wellbeing  (objective function) | Determined by the mean strength of the agent’s relationships (mean trust) | range 1 – 5 (1-10 in N=500 simulations) |
| All: Alliance | Determined by the mean strength of the agent’s relationships (mean trust) modulated by total number of relationships | as above |
| RS: Risk | Mean trust divided by the interaction frequency | as above |
| ST: Stress | Determined by the number of relationships | as above |
| R: Resource | Determined by the number of forage turns times number of relationships | as above |
| F:S ratio | Proportion of Foraging to Social interaction turns for each agent. | 1: 8 initially allowed to vary by the mutation rate |
| Fitness | Positive objective functions (WB+All+R) – negative objective functions (RS +ST) | Calculated for each agent at the end of each cycle |
| Selection/breeding rate | % of the population which was removed and replaced each cycle, ranked by fitness | 20% (also 5, 10%) |
| Mutation rate | Randomly selected parameter change for F:S and Reject ratios | +/- 1 |

**Supplementary References**

Abbott, DH, Keverne, EB, Moore, GF, & Yodyinguad, U. 1986. Social suppression of reproduction in subordinate talapoin monkeys, *Miopithecus talapoin*. In: J. Else & P.C. Lee (eds) Primate Ontogeny, pp. 329-341. Cambridge: Cambridge University Press

Dunbar, RIM. 1980. Determinants and evolutionary consequences of dominance among female gelada baboons. Behavioural Ecology and Sociobiology 7: 253-265.

Dunbar, RIM. 1988. Habitat quality, population dynamics and group composition in colobus monkeys (*Colobus guereza*). International Journal of Primatology 9: 299-329.

Hill, RA, Lycett, J. & Dunbar, RIM. 2000. Ecological determinants of birth intervals in baboons. Behavioral Ecology 11: 560-564.
